# Epigenetic reactivation of estrogen receptor-α (ERα) by genistein enhances hormonal therapy sensitivity in ERα-negative breast cancer

**DOI:** 10.1186/1476-4598-12-9

**Published:** 2013-02-04

**Authors:** Yuanyuan Li, Syed M Meeran, Shweta N Patel, Huaping Chen, Tabitha M Hardy, Trygve O Tollefsbol

**Affiliations:** 1 Department of Biology, University of Alabama at Birmingham, Birmingham, Alabama, USA; 2 Center for Aging, University of Alabama at Birmingham, Birmingham, Alabama, USA; 3 Comprehensive Cancer Center, University of Alabama at Birmingham, Birmingham, Alabama, USA; 4 Nutrition Obesity Research Center, University of Alabama at Birmingham, Birmingham, Alabama, USA; 5 Comprehensive Diabetes Center, University of Alabama at Birmingham, Birmingham, Alabama, USA; 6 School of Medicine, University of Alabama at Birmingham, Birmingham, Alabama, USA; 7 Endocrinology Division, Central Drug Research Institute, Lucknow, India

**Keywords:** Genistein, ERα, Tamoxifen, Epigenetic, Breast cancer

## Abstract

**Background:**

Estrogen receptor-α (ERα)-negative breast cancer is clinically aggressive and normally does not respond to conventional estrogen target-directed therapies. The soybean isoflavone, genistein (GE), has been shown to prevent and inhibit breast cancer and recent studies have suggested that GE can enhance the anticancer capacity of an estrogen antagonist, tamoxifen (TAM), especially in ERα-positive breast cancer cells. However, the role of GE in ERα-negative breast cancer remains unknown.

**Methods:**

We have evaluated the *in vitro* and *in vivo* epigenetic effects of GE on *ERα* reactivation by using MTT assay, real-time reverse transcription-polymerase chain reaction (RT-PCR) assay, western-blot assay, immunoprecipitation (ChIP) assay, immunohistochemistry and epigenetic enzymatic activity analysis. Preclinical mouse models including xenograft and spontaneous breast cancer mouse models were used to test the efficacy of GE *in vivo*.

**Results:**

We found that GE can reactivate *ERα* expression and this effect was synergistically enhanced when combined with a histone deacetylase (HDAC) inhibitor, trichostatin A (TSA), in ERα-negative MDA-MB-231 breast cancer cells. GE treatment also re-sensitized ERα-dependent cellular responses to activator 17β-estradiol (E_2_) and antagonist TAM. Further studies revealed that GE can lead to remodeling of the chromatin structure in the *ERα* promoter thereby contributing to *ERα* reactivation. Consistently, dietary GE significantly prevented cancer development and reduced the growth of ERα-negative mouse breast tumors. Dietary GE further enhanced TAM-induced anti-cancer efficacy due at least in part to epigenetic *ERα* reactivation.

**Conclusions:**

Our studies suggest that soybean genistein can epigenetically restore *ERα* expression, which in turn increases TAM-dependent anti-estrogen therapeutic sensitivity *in vitro* and *in vivo*. The results from our studies reveal a novel therapeutic combination approach using bioactive soybean product and anti-hormone therapy in refractory ERα-negative breast cancer which will provide more effective options in breast cancer therapy.

## Background

Breast cancer is the most common type of cancer and the second leading cause of death among women in the United States. The principle therapeutic strategy for breast cancer involves surgical removal of the primary tumor following extensive radiotherapy and chemotherapy. Several clinical trials have suggested that estrogen ablation or anti-estrogen strategy is effective in the prevention or treatment of breast cancer, especially in estrogen receptors (ERs)-dependent breast cancer [1-3]. There are two major isoforms of ERs (ERα and ERβ) that have been identified and the ERα isoform is believed to primarily contribute to estrogen-induced growth stimulatory effects in breast cancer [4]. Estrogens binding to ERs result in activated signaling pathways leading to cellular proliferation and differentiation in normal mammary tissue. However, aberrant activation of estrogen-ER signaling renders unlimited and uncontrolled cell proliferation which occurs in most breast tumors [5-7]. The estrogen antagonist, tamoxifen (TAM), is currently the first-line medical treatment for ERα-positive breast cancer at all stages of this disease in both pre- and postmenopausal women [8]. TAM has also been shown to have potential benefit for the prevention of breast cancer among women at high risk of breast cancer [1]. However, ERα-negative breast cancers do not respond to TAM treatment and generally have a more clinically aggressive progression resulting in a poorer prognosis [9].

Extensive studies have shown that the major cause for inactive ERα signaling is the absence of *ERα* gene expression. Although the precise mechanisms of *ERα* transcription regulation are still under investigation, it has been clear that acquired loss of *ERα* transcription rather than a genetic alteration such as DNA mutations is a potential mechanism for hormone resistance in ERα-negative breast cancer [10]. Recent studies indicate that epigenetic mechanisms, which primarily involve two pathways, DNA methylation and histone modification, may play a crucial role in regulating *ERα* expression [11-14]. Supportive evidence has included intervention application of epigenetic modulators such as DNA methyltranferase (DNMT) inhibitor, 5-aza-2’-deoxycytidine (5-aza), and histone deacetylase (HDAC) inhibitor, trichostatin A (TSA), which successfully induced *ER* expression and sensitized hormone-resistant ERα-negative breast cancer cells to chemotherapy [13-16]. In this regard, it is increasingly evident that epigenetic events play an important role in *ERα* gene expression.

Despite a high incidence and mortality by breast cancer in the United States and Europe, Asian women who consumed 20–50 times more soy products per capita than their western counterparts have much less susceptibility to developing breast cancer [17-19]. Soybean product is a rich source of genistein isoflavone, which is believed to be a potent botanical chemopreventive compound against various types of cancers, including breast cancer [20]. Genistein (GE) exerts its anti-cancer properties through various mechanisms such as anti-oxidation, induction of apoptosis and differentiation as well as inhibition of angiogenesis and proliferation [21-24]. One potential mechanism that has recently received considerable attention is that GE may regulate gene transcription by modulating epigenetic events [25-27]. This hypothesis is supported by studies showing that dietary GE causes epigenetic changes in mouse prostate [28]. Our studies as well as others have also suggested an epigenetic associated-prevention role of GE by regulating key tumor-related genes such as *p16*^*INK4a*^ and the human telomerase reverse transcriptase (*hTERT*) gene, leading to tumor prevention and suppression in malignant human mammary cells [26,29]. More importantly, studies have shown that GE treatment can enhance or sensitize the preventive and inhibitory effects of TAM in ERα-positive breast cancer cells [30,31]. However, the potential impact of GE on the estrogen-ERα pathway and the further combination effect of GE with TAM on ERα-negative breast cancer have not been well defined experimentally. Since TAM is widely used for prevention and treatment for breast cancer and soy products are recognized as important bioactive components against breast cancer, it is imperative to define the interactive effect between soy components and TAM on breast cancer prevention, especially on intractable hormone-resistant breast cancer.

We therefore hypothesize that GE might epigenetically reactivate ERα which may facilitate TAM-mediated estrogen-dependent therapy by resensitizing ERα-negative breast cancer cells. Our studies used both *in vitro* and *in vivo* approaches to investigate the epigenetic effects of soybean GE on *ERα* reactivation and how this change may affect cell sensitivity to conventional anti-hormone agents such as TAM in hormone-resistant breast cancer. Our findings help to develop a novel combination approach by using soybean product and hormone antagonists for chemoprevention and therapeutic strategies in estrogen-resistant breast cancers.

## Materials and methods

### Cell culture and cell treatment

Breast cancer cell lines including ERα-positive MCF-7 and ERα-negative MDA-MB-231 and MDA-MB-157 cells as well as normal human mammary epithelial cells (HMECs) were obtained from American Type Culture Collection (ATCC) and Lonza (Basel, Switzerland), respectively. Breast cancer cells were grown in phenol-red–free medium DMEM (Invitrogen, Carlsbad, CA) supplemented with 10% dextran-charcoal–stripped fetal bovine serum (Atlanta Biologicals, Lawrenceville, GA) and 1% penicillin/streptomycin (Mediatech, Herndon, VA). HMECs were grown in serum-free Mammary Epithelial Growth Medium (MEGM) without sodium bicarbonate accompanied with MEGM SingleQuots (Lonza) at 37°C and 0.1% CO_2_. Breast cancer cells were maintained in a humidified environment of 5% CO_2_ and 95% air at 37°C. To evaluate ERα expression, attached MDA-MB-231 and MDA-MB-157 cells were treated with various concentrations of genistein (GE) (Sigma, St. Louis, MO) for 3 days while MCF-7 cells served as a positive control. The medium with GE was replaced every 24 h for the duration of the experiment. Control cells received equal amounts of DMSO (Sigma) in the medium. For the combination study, cells were treated with an optimal concentration (25 μM) of GE based on our results and 5-aza (2 μM for 2 days) (Sigma) or TSA (100 ng/ml for 12 h) (Sigma) alone or together for a total 3 days as common recommended doses of these compounds [32]. HMECs were used as a normal control to evaluate potential toxicity in response to GE and/or TSA treatment. To observe the effects of 17β-estradiol (E_2_) (Sigma) and tamoxifen (TAM) (Sigma) on *ERα* expression, GE and/or TSA-pretreated MDA-MB-231 cells [GE at 25 μM for 3 days or TSA at 100 ng/ml for 12 h for single treatment, and GE (25 μM for 2 days) + TSA (100 ng/ml for 12 h) for combination treatment] were then exposed with or without 10 nM of E_2_ or 1 μM TAM for an extra two days, respectively.

### MTT assay for cell viability

To determine the effects of GE alone or in combination with TSA on cell viability when exposed with E_2_ or TAM, aliquots of 5 × 10^3^ MCF-7 and MDA-MB-231 cells were seeded in triplicate in 96-well plates and treated with the indicated compounds as described above. MTT solution was added to the medium to achieve a final concentration of 1 mg/ml. The cells were incubated at 37°C and dissolved in 100 μl DMSO after 4 h incubation. The absorbance of the cell lysates in DMSO solution was read at 570 nm by a microplate reader (Bio-Rad, Hercules, CA).

### RNA interference

Validated siRNA for *ERα* and the appropriate control RNAi (Applied Biosystems) were transfected into MDA-MB-231 cells using the Silencer siRNA Transfection II Kit (Applied Biosystems) according to the protocols provided by the manufacturer. Real-time PCR assay was performed to verify the result of *ERα* gene knockout.

### Dietary preparation

Two designed diets were used in this study: control diet (phytoestrogen-free modified AIN-93G diet with 7% corn oil substituted for 7% soybean oil; TD. 95092; Harlan Teklad, Madison, WI) and GE diet (modified AIN-93G diet supplemented with 250 mg/kg genistein; TD. 00417; Harlan Teklad) [33]. The level of GE in this diet results in the animals being exposed to concentrations comparable with those received by humans consuming high-soy diets [34]. Harland Teklad supplied all diet ingredients except GE powder obtained from LKT Laboratories, St. Paul, MN.

### Animal models

We have used two mouse models such as the orthotopic breast cancer mouse model (treatment model) and spontaneous breast cancer mouse model (prevention model) in this study. Virgin female immunodeficiency Nu/Nu Nude mice (Crl:NU-*Foxn1nu*) were used for xenograft breast cancer study. Nude mice at 4–6 weeks of age were obtained from Charles River Laboratories (Wilmington, MA). The C3(1)-SV40 Tag transgenic mouse model [FVB-Tg(C3-1-TAg)cJeg/JegJ] was used for prevention model since they can spontaneously develop breast tumors at early ages (around 15–20 wks) [35]. The C3(1)-SV40 Tag breeder mice at 4 wks were obtained from Jackson Laboratory (Bar Harbor, ME) and mice colonies were maintained in our laboratory. All the mice were housed in the Animal Resource Facility of the University of Alabama at Birmingham and were maintained under the following conditions: 12-h dark/12-h light cycle, 24 ± 2°C temperatures, and 50 ± 10% humidity.

### Animal experimental designs

#### Protocol 1. Tumor xenografts assay for treatment effects of GE

After one week of acclimatization, Nu/Nu Nude mice were randomly divided into four groups (5 mice each) and administered either control or GE diet as described above. Diets were provided from two weeks prior to injection and the mice continued to receive the corresponding experimental diets throughout the study.

To determine the *in vivo* efficacy of GE on ERα reactivation and subsequent chemosensitization to estrogen antagonist, TAM, in human ERα-negative breast tumor xenografts, exponentially growing MDA-MB-231 cells were mixed at a 1:1 ratio with Matrigel (Becton Dickinson). A 100 μl suspension containing 1 × 10^6^ cells was injected orthotopically into the mammary fat pad of each mouse.

The experimental groups were as follows: Group (1). Control group: Mice were fed with control diet as described previously; Group (2). GE group: Mice were fed with GE diet (250 mg/kg, equal amount of maximal genistein uptake from daily diet); Group (3). TAM group: Mice were fed with control diet plus TAM treatment for 3 wks after two wks of post-injection (25 mg/pellet with 21 days release, subcutaneous implantation under the neck area, Innovative Research of America, Aarasota, FL); Group (4). GE + TAM group: Mice were fed a GE diet and received TAM treatment as described above.

#### Protocol 2. Spontaneous breast cancer mouse model for preventive effects of GE

The C3(1)-SV40 Tag transgenic mouse model was used for prevention study of GE treatment because this mouse model can spontaneously develop breast cancer. More importantly, this model tends to develop hormone-independent invasive breast cancer (ERα-negative breast cancer), which is perfectly suitable to our investigation purpose for *ERα* reactivation. The *Tag* genotypes were identified at 21 days of life by analysis of tail DNA using standard PCR techniques according to previous studies [35]. The C3(1)-SV40 Tag mice at 4–6 weeks of age were randomly divided to different experimental groups (10 mice/group) and control and GE diets were administered at the indicated time and the diets were continued throughout the study.

The experimental groups were as follows: Group (1). Control group: Mice were fed control diet as described previously; Group (2). GE group: Mice were fed GE diet as described previously; Group (3). TAM group: Mice were fed control diet and TAM tablet was implanted subcutaneously for 3 wks when tumor size reaches ~400 mm^3^; (4). GE + TAM group: Mice were administered with GE diet and TAM treatment as described above.

#### Tumor parameters monitoring, experimental endpoint and tissue sample collection

Tumor diameters and body weight were measured weekly. Tumor volumes were measured by a caliper and estimated using the following formula: tumor volume (cm^3^) = (length × width^2^) × 0.523 [31]. For *Protocol 1.*, the experiment was finished when the mean of tumor diameter in the control mice exceeded 1.0 cm following the guidelines of Institutional Animal Care and Use Committee at the University of Alabama at Birmingham. As to *Protocol 2.*, the first palpable tumor was used to calculate tumor latency for mice that developed either single or multiple mammary tumors. Mice were sacrificed when the mean of tumor diameter of the biggest tumor exceeded 1.5 cm and all mice were euthanized at 25 wks regardless of tumor size. At the end of the experiment, the mice were sacrificed, primary tumors were excised and weighed. A tumor slice from each primary tumor tissue was carefully dissected and fixed in 10% buffer-neutralized formalin for histology and immunohistochemistry. Tumor specimens were snap frozen in liquid nitrogen for further studies such as RNA and protein extraction. All procedures with animals were reviewed and approved by the Institutional Animal Care and Use Committee at the University of Alabama at Birmingham.

### Quantitative real-time PCR

Both ERα-positive MCF-7 and ERα-negative MDA-MB-231 and MDA-MB-157 cells were cultured and treated as described above. Total RNA from cells or mice tumor tissues was extracted using the RNeasy kit (Qiagen, Valencia, CA) according to the manufacturer’s instructions. Genes of interest were amplified using 1 μg of total RNA reverse transcribed to cDNA using the Superscript II kit (Invitrogen) with oligo-dT primer. In the real-time PCR step, PCR reactions were performed in triplicate and primers specific for *ERα*, *progesterone receptor* (*PGR*), *DNA methyltransferase* (*DNMT*), *histone deacetylase* (*HDAC*) and *glyceraldehyde-3-phosphate dehydrogenase* (*GAPDH*) provided by Inventoried Gene Assay Products (Applied Biosystems, Foster City, CA) were used for Platinum Quantitative PCR Supermix-UDG (Invitrogen) in a Roche LC480 thermocycler. Thermal cycling was initiated at 94°C for 4 min followed by 35 cycles of PCR (94°C, 15 s; 60°C, 30 s). GAPDH was used as an endogenous control, and vehicle control was used as a calibrator. The relative changes of gene expression were calculated using the following formula: fold change in gene expression, 2^-ΔΔCt^ = 2^-{ΔCt (treated samples) - ΔCt (untreated control samples)}^, where ΔCt = Ct (test gene) - Ct (GAPDH) and Ct represents threshold cycle number.

### Western blot analysis

For western blot analysis, protein extracts were prepared by RIPA Lysis Buffer (Upstate Biotechnology, Charlottesville, VA) according to the manufacturer’s protocol. Proteins (50 μg) were electrophoresed on a 10% SDS-polyacrylamide gel and transferred onto nitrocellulose membranes. Membranes were probed with antibodies to ERα (Ab-12; NeoMarkers, Fremont, CA), HDAC1 (H11; Santa Cruz Biotechnology) and DNMT1 (ab 13537; Abcam, San Francisco, CA ) respectively, then each membrane was stripped with and reprobed with beta-actin antibody (13E5, Cell Signaling Technology, Boston, MA) as loading control. Molecular weight markers were run on each gel to confirm the molecular size of the immunoreactive proteins. Immunoreactive bands were visualized using the enhanced chemiluminescence detection system (Santa Cruz Biotechnology) following the protocol of the manufacturer.

### Immunohistochemical determination of tumor cell proliferation and ERα expression

Tumor sections (5 μm thick) were deparaffinized and rehydrated in a series of graded alcohols. Following rehydration, an antigen retrieval process was performed by placing the slides in 10 mmol/L sodium citrate buffer (pH 6.0) at 95°C for 20 min followed by 20-min cooling at room temperature. The sections were washed in PBS and nonspecific binding sites were blocked with 1% bovine serum albumin with 2% goat serum in PBS before incubating with either anti-proliferating cell nuclear antigen (PCNA) (Cell Signaling Technology) or anti-ERα antibody for 2 h at room temperature. After washing with PBS, the sections were incubated with biotinylated secondary antibody for 45 min followed by horseradish peroxidase-conjugated streptavidin, washed in PBS, incubated with diaminobenzidine substrate, and counterstained with hematoxylin. Photographs of representative pictures were taken and the numbers of PCNA-positive or ERα-positive cells were detected and counted using a light microscope. The results are presented as the number of positive cells × 100 divided by the total number of cells.

### Chromatin Immunoprecipitation (ChIP) Assay

MDA-MB-231 cells were treated with 25 μM GE and 100 μg/ml TSA alone or in combination for the indicated times. Approximately 2 × 10^6^ cells were cross-linked with a 1% final concentration of formaldehyde (37%, Fisher Chemicals, Fairlawn, NJ) for 10 min at 37°C. ChIP assays were performed with the EZ Chromatin Immunoprecipitation (EZ ChIP^TM^) assay kit according to the manufacturer’s protocol (Upstate Biotechnology) as described previously [29,32]. The epigenetic antibodies used in the ChIP assays were ChIP-validated acetyl-histone H3 (Upstate Biotechnology), acetyl-histone H3-Lys9 (H3K9) (Upstate Biotechnology), acetyl-histone H4 (Upstate Biotechnology), dimethyl-histone H3-Lys4 (H3K4) (Upstate Biotechnology), histone deacetylase1 (HDAC1) (Santa Cruz Biotechnology) and DNMT1 (Abcam, Cambridge, MA). ChIP-purified DNA was amplified by standard PCR using primers specific for the *ERα* promoter ranging from +78 to +227 in exon 1 and yielding a 150 bp fragment: sense, 5’-GAACCGTCCGCAGCTCAAGATC-3’ and anti-sense, 5’- GTCTGACCGTAGACCTGCGCGTTG -3’. PCR amplification was performed using the 2×PCR Master Mix (Promega, Madison, WI) and the reaction was initiated at 94°C for 4 min followed by 30 cycles of PCR (94°C, 30 s; 56°C, 30 s; 72°C, 1 min), and extended at 72°C for 5 min. After amplification, PCR products were separated on 1.5% agarose gels and visualized by ethidium bromide fluorescence using Kodak 1D 3.6.1 image software (Eastman Kodak Company, Rochester, NY). Quantitative data were analyzed using the Sequence Detection System software version 2.1 (PE Applied Biosystems, Foster City, CA).

### HDACs and DNMTs activity assay

Nuclear protein from cultured MDA-MB-231 cells and breast tumor tissues was extracted by using the nuclear extraction reagent (Pierce, Rockford, IL). The activities of HDACs (Active Motif, Carlsbad, CA) and DNMTs (Epigentek, Brooklyn, NY, USA) were performed according to the manufacturer’s protocols as reported previously [32,36]. The enzymatic activities of HDACs and DNMTs were detected by using a microplate reader at 450 nm.

### Statistical analyses

Microscopic immunohistochemical analysis of tissue sections was performed using an Olympus BX41 microscope fitted with a Q-color 5 Olympus camera. Results from Real-time PCR and ChIP assays were derived from at least three independent experiments. For quantification of ChIP products, Kodak 1D 3.6.1 image software was used. The protein levels were quantified by optical densitometry using ImageJ Software version 1.36b (http://rsb.info.nih.gov/ij/). Statistical significance between treatment and control groups was evaluated by one-way ANOVA followed by Tukey’s test for multiple comparisons by using GraphPad Prism version 5.00 for Windows, GraphPad Software (http://www.graphpad.com). Tumor-free intervals (tumor latency) for survival curves were calculated using the Mantel-Cox proportional model and differences were tested using the log-rank statistic. Values were presented as mean ± SD and *P* < 0.05 was considered significant.

## Results

### Combination treatment with GE and TSA synergistically reactivated ERα expression in ERα-negative breast cancer cells

**Figure 1:**
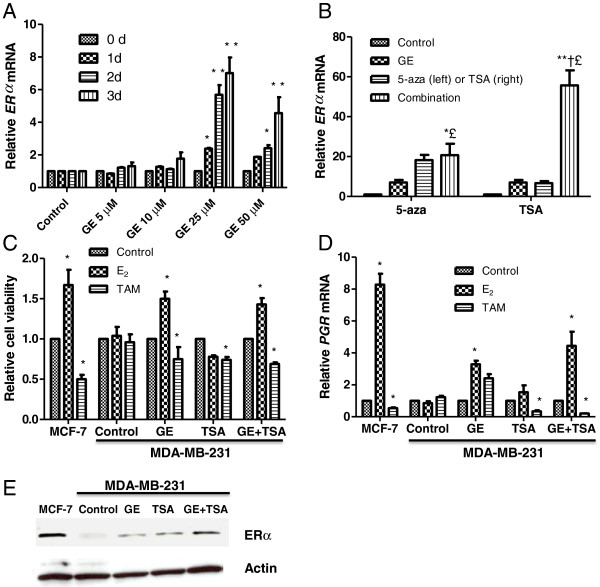
**GE and TSA synergistically induced *ERα *re-expression in ERα-negative MDA-MB-231 breast cancer cells. A**) Graphic presentation of dose- and time- dependent *ERα *expression by GE treatment. MDA-MB-231 cells were plated in 6-well plates in triplicate and exposed to various concentrations of GE for up to 3 days. **B**) *ERα* expression changes by the combined treatment of GE with 5-aza (left) and TSA (right). The MDA-MB-231 cells were treated with or without either 25 μM GE or 2 μM 5-aza and 100 ng/ml TSA alone or together for 3 days. Control cells were grown in parallel with the treated cells but received vehicle DMSO. Quantitative real-time PCR was performed to measure relative transcription of *ERα*. **C**) Cellular viability in response to E_2_ and tamoxifen (TAM). **D**) The expression of *PGR*, an ERα target gene, in response to E_2_ and tamoxifen. GE and/or TSA-pretreated MDA-MB-231 cells were treated with or without 10 nM of E_2 _or 1 μM TAM for 1 days. MCF-7 cells served as a positive control. Cells were harvested at the indicated time periods and assessed for cellular viability and *PGR* expression, respectively. Cellular viability was measured by MTT assay and *PGR* expression was detected by quantitative real-time PCR. Data are in triplicate from three independent experiments and were normalized to *GAPDH* and calibrated to levels in the relevant control samples. Bars, SD; *, *P* < 0.05, * * *P *< 0.001, significantly different from control; £, *P *< 0.05, significantly different from GE (Figure 1B); †, *P *< 0.05, significantly different from 5-aza or TSA (Figure 1B). **E**) The ERα protein levels were determined by western-blot analysis. MCF-7 cells served as a positive control. GAPDH antibody was used to ensure equal loading. Representative photograph from an experiment was repeated three times.

Our previous studies have shown that (−)-epigallocatechin-3-gallate, an active component in green tea polyphenols, can induce *ERα* re-expression in ERα-negative breast cancer cells [32]. We hypothesize that dietary GE may have a similar effect on *ERα* expression since both compounds are considered to exert their anticancer properties via epigenetic control. We initiated our study to determine whether GE can impact *ERα* expression and the optimal dose and time point that will induce *ERα* activation. We treated ERα-negative breast cancer cells, MDA-MB-231, with various concentrations of GE at different time points and observed *ERα* transcription under these treatments. As shown in Figure 1A, a significant increase of *ERα* transcription (*p*<0.001) was observed with 25 μM of GE and the *ERα* reactivation was predominant at 3 days of treatment. This GE concentration is considered to be equivalent to the maximal consumption of soybean product per day or a pharmaceutically available GE supplementary tablet, suggesting a potential bioavailability of this treatment. This result indicates that treatment with 25 μM GE at 3 days could serve as an optimal condition in regulating *ERα* re-expression in ERα-negative breast cancer cells.

We also tested combination effects of GE with other epigenetic modulators such as the histone deacetylase (HDAC) inhibitor, trichostatin A (TSA), and a demethylation agent, 5-aza-2’-deoxycytidine (5-aza), on *ERα* re-expression because epigenetic mechanisms such as histone modifications and DNA methylation were known to contribute to *ERα* regulation. Both TSA and 5-aza have been reported to successfully activate *ERα* transcription in human ERα-negative breast cancer cells [13], but have not previously been combined with GE in ER studies. Consistent with previous studies, our results indicated that 5-aza and TSA alone reactivated *ERα* expression in MDA-MB-231 cells. More importantly, we found that the combined treatment of GE and TSA induced a significant synergistic effect on *ERα* re-expression, much more so than GE in combination with 5-aza (Figure 1B). This effect was further confirmed by the results of ERα protein levels in Figure 1E showing that combination treatment using GE and TSA led to more abundant *ERα* re-expression than the other treatments administered alone.

To further verify the GE effects on *ERα* reactivation on an ERα-negative breast cancer cell line other than MDA-MB-231 cells, we performed similar experiments on ERα-negative MDA-MB-157 cells (Additional files 1A and 1B). We found a dose-dependent effect of *ERα* up-regulation in response to GE treatment and combination treatment of 25 μM of GE with TSA but not 5-aza resulted in a synergistic effect on *ERα* reactivation. This similar response to GE treatment as seen in MDA-MB-231 cells suggests that this combination regimen results in a prevalent effect on *ERα* reactivation in different ERα-negative breast cancer cells as well. In Additional file 1C, we also evaluated the potential toxicity of this novel combination in normal human mammary epithelial cells (HMECs) and found that neither of these two compounds acting alone nor in combination caused inhibitory effects on cell viability in HMECs cells indicating the combined treatment of GE and TSA is potentially safe and may apply for *in vivo* studies.

Our results reveal a novel combination regimen by using a bioactive compound, GE, and an HDAC inhibitor, TSA, in converting ERα status which may provide a promising therapeutic strategy especially in ERα-negative breast cancer. These results also indicate a more important role of histone modification rather than DNA methylation in GE induced-*ERα* reactivation.

### GE and TSA re-sensitized ERα-negative breast cancer cells to E_2_ and TAM

In the presence of ER, a series of ER-dependent cellular responsiveness is stimulated including cellular proliferation and downstream ER-response gene expression by binding ER with hormone signals such as 17β-estradiol (E_2_) [4,5]. This effect could be blocked by the E_2_ antagonist, tamoxifen (TAM), leading to cell growth arrest by competing with E_2_ binding to ER [8]. Since our aforementioned findings suggested that GE combined with TSA led to synergistic re-expression of *ERα* mRNA in ERα-negative breast cancer cells, we therefore sought to investigate whether this re-expression of *ERα* could effectively respond to E_2_ and TAM treatments. We investigated the changes in cellular viability as well as the expression of the *ERα*-responsive downstream gene, *progesterone receptor* (*PGR*), in response to E_2_ or TAM, with treatments of GE and TSA alone or together in ERα-negative MDA-MB-231 breast cancer cells. ERα-positive MCF-7 breast cancer cells served as a positive control. As shown in Figures 1C and 1D, MCF-7 cells showed a significant response to E_2_ and TAM, whereas untreated MDA-MB-231 cells have no response to these two compounds with respect to cell growth and *PGR* expression. Treatments with either GE or TSA alone induced a partial response to E_2_ and TAM. In particular, GE treatment alone led to a positive response in cell growth but not in *PGR* expression, whereas TSA acting alone caused *PGR* response but not in cell growth in response to E_2_ and TAM, which is likely due to the limited increased level of *ERα* re-expression with treatment of GE and TSA alone. Eventually, combined treatments with GE and TSA resulted in significant changes in cellular growth and downstream *PGR* expression in response to E_2_ and TAM in ERα-negative MDA-MB-231 cells in a similar manner to that observed in ERα-positive MCF-7 cells (Figures 1C and 1D).

We also performed RNAi experiments to further test whether *ERα* presence plays an important role in GE and/or TAM-induced cellular growth inhibition in ERα-negative MDA-MB-231 breast cancer cells. As shown in Additional file 2A and 2B, GE alone or with TAM treatment resulted in a significant inhibition of cellular viability compared to these two treatments with silencing expression of *ERα*. These results suggest that reactivated *ERα* potentiates the efficacy of GE and TAM against ERα-negative breast cancer cells.

Our results indicate that the combination of GE and TSA can induce functional *ERα* re-activation and re-sensitize ERα-negative breast cancer cells to E_2_ activator and TAM antagonist. This novel combination could provide an important clinical implication in future alternative therapeutic strategies for hormone-resistant breast cancer.

### GE and TSA led to histone modification changes in the ERα promoter

**Figure 2:**
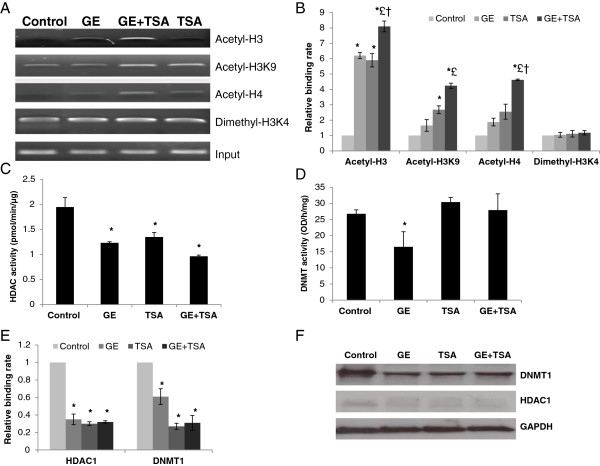
**Epigenetic alterations in response to GE and/or TSA treatments. **(**A**) Histone modification patterns in the *ERα *promoter were analyzed by ChIP assay. Representative photograph from an experiment was repeated in triplicate. (**B**) Histone modification enrichment in the *ERα* promoter was calculated from the corresponding DNA fragments amplified by ChIP-PCR as shown above. MDA-MB- 231 cells were treated as described previously and analyzed by ChIP assays using chromatin markers including acetyl-H3, acetyl-H3K9, acetyl-H4, dimethyl-H3K4 and mouse IgG control in the promoter region of *ERα*. Inputs came from the total DNA and served as the same ChIP-PCR conditions. DNA enrichment was calculated as the ratio of each bound sample divided by the input while the untreated MDA-MB-231 control sample is represented as 1. (**C**) HDACs enzymatic activity. (**D**) DNMTs enzymatic activity. Nuclear proteins of MDA-MB-231 cells were extracted after the treatment as described above. The HDACs and DNMTs activity assays were performed according to the manufacturer’s protocols. (**E**) Binding abilities of HDACs and DNMTs in the *ERα *promoter were determined by ChIP assay as described previously. The values of enzymatic activities of HDACs and DNMTs are the means of three independent experiments. Columns, mean; Bars, SD. *, *P* < 0.05, significantly different from control; £, *P *< 0.05, significantly different from GE; †, *P *< 0.05, significantly different from TSA. (**F**) The protein level changes of HDACs and DNMTs were determined by western-blot analysis. GAPDH antibody was used to ensure equal loading. Representative photograph from an experiment was repeated three times.

GE has been reported to influence gene expression via epigenetic mechanisms and *ERα* expression is frequently mediated by epigenetic controls. Therefore, we focused on our subsequent experiments to investigate whether GE may affect histone remodeling on the *ERα* gene. We tested several chromatin markers, for example, acetyl-H3, acetyl-H3K9, acetyl-H4 and dimethyl-H3K4, to explore enrichment changes of these markers that may affect *ERα* gene expression in response to GE in MDA-MB-231 cells. We found that GE treatment can increase enrichment of three histone acetylation chromatin markers, acetyl-H3, acetyl-H3K9, acetyl-H4 (especially in the histone H3 molecule, *P* < 0.05), and slightly increased one histone methylation chromatin marker, dimethyl-H3K4 (Figures 2A and 2B). The abundance of these chromatin markers indicates a loosening chromatin structure leading to active gene transcription. In addition, histone remodeling changes were more prominent when GE was combined with TSA than either treatment alone, which is consistent with our aforementioned findings. Our results indicate that GE and TSA treatment results in a strengthened *ERα* expression that might be due to enhanced histone remodeling of the *ERα* gene induced by this combination.

### Epigenetic enzymes changes in response to GE

To further interpret the mechanisms of epigenetic modulations on GE-induced *ERα* re-expression in ERα-negative breast cancer cells, we assessed two important epigenetic enzymatic activities such as HDACs and DNMTs. As shown in Figure 2C, both GE and TSA alone can significantly reduce HDACs activity, while their combination led to a more prominent reduction than any compound acting alone. As to DNMTs activity shown in Figure 2D, only GE treatment caused a significant inhibition suggesting that GE and TSA-induced *ERα* reactivation may be primarily mediated through histone remodeling rather than DNA methylation. We also found that GE caused a reduction of binding to the *ERα* promoter as well as gene expression for both HDACs and DNMTs (Figures 2E and 2F). The different DNMTs enzymatic activities and protein expression in response to GE and/or TSA treatment suggest that DNMT1 may affect *ERα* expression through transcription regulation rather than directly influencing DNA methylation status in the *ERα* promoter, which has been confirmed by further bisulfite sequencing analysis on the *ERα* promoter (data not shown). Although GE alone and combination treatment also inhibited DNMTs binding and its expression, it might lead to DNMT-involved transcriptional repressor recruitment blocking which also contributes to *ERα* re-expression [37]. These results indicate that GE alone affects *ERα* expression most likely via both epigenetic pathways involving histone modification and DNA methylation, whereas, when GE is combined with TSA, a synergistic effect of *ERα* reactivation is induced by a more efficient epigenetic response to histone modification rather than DNA methylation. Taken together, our results further indicate that GE can restore *ERα* expression in ERα-negative breast cancer cells through influencing epigenetic mechanisms and this effect is strengthened in the presence of TSA, a deacetylation inhibitor.

### Dietary GE inhibited the growth of breast cancer and increased therapeutic sensitivity of TAM in ERα(−) breast cancer xenografts

**Figure 3:**
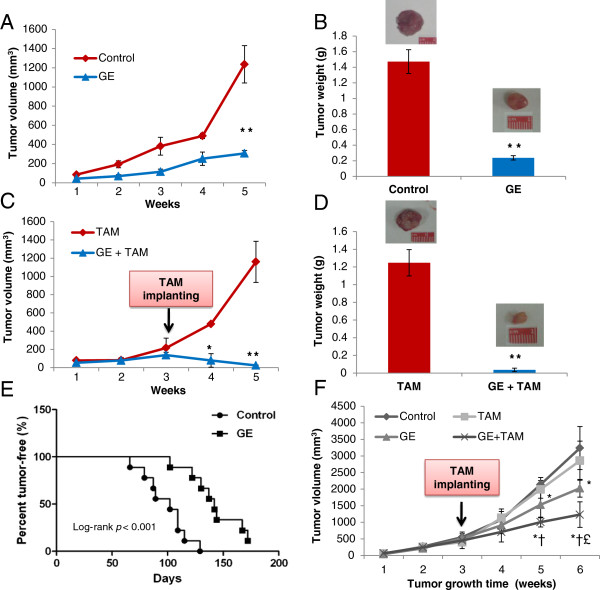
**Breast tumor growth in mouse models by dietary GE and/or TAM treatments.** Two mouse models were used in this study. Figures 3A, 3B, 3C and 3D are involved in orthotopic breast cancer mouse model (*Protocol 1*, seen in Materials and methods). Female athymic nude mice were injected with MDA-MB-231 cells. GE or control diets were provided from two weeks prior to injection and one 21-day release of 25 mg TAM pellet was implanted subcutaneously two wks post-injection. **A**) and **B**) GE alone inhibited the growth of mice xenografts. **C**) and **D**) GE re-sensitized TAM in tumor suppression. **A**) and **C**) Tumor volume during the experiment. **B**) and **D**) tumor xenograft tissues were harvested at the termination of the experiment. Figures 3E and 3F are spontaneous breast cancer mouse model (*Protocol 2*). Diets were administered to C3(1)-SV40 Tag transgenic mice at 4–6 wks of age and TAM treatments were performed when tumor volumes reaches to ~400 mm^3^. **E**) Dietary GE increased the latency of tumor development. **F**) Tumor volume changes after TAM implantation. Tumor volumes were calculated by using the formula: volume (mm^3^) = (length × width^2^) × 0.523, and represented as mean ± SD (mm^3^) for each group. Tumor weight is the wet weight of the tumor per mouse in each group and is reported as mean ± SD (g). The actual tumor images were selected to represent the difference of tumor sizes and a ruler was included for tumor measurement. Symbols and columns, mean; Bars, SD from 5 or 10 mice per group; * *p *< 0.01, **, *p *< 0.001 significantly different from control group; †, *P *< 0.05, significantly different from TAM group (Figure 3F); £, *P *< 0.05, significantly different from GE (Figure 3F).

As we have found that GE treatment led to functionally *ERα* reactivation in ERα-negative breast cancer cells *in vitro*, we sought to determine whether dietary administration of GE can inhibit the growth of ER(−) breast cancer through combining with anti-hormone therapy such as TAM *in vivo*. ERα-negative breast cancer cells, MDA-MB-231, were used to grow xenografts in athymic nude mice that had been fed a diet supplemented with GE for two weeks before injection of the tumor cells and continued throughout the study. We have not found any differences in the daily consumption of diet and drinking water by the mice among the different groups and the mice that were given the GE diet (250 mg/kg) did not exhibit any physical sign of toxicity (data not shown). Previous studies also have shown that administration of GE in the diet at this concentration is equivalent to the maximal consumption of soybean products [34]. Asian women who consume soybean food as their primary daily diet show low incidence of breast cancer suggesting protective effects of this diet [18,19,38]. Periodic measurement of the tumor volume indicated that the average tumor growth in terms of total tumor volume per mouse in the control group was dramatically increased compared with the GE-treated group (Figure 3A). In addition, in the group of mice that received the GE diet, the overall tumor growth rate was inhibited and the tumor volume at the termination of the experiment was significantly reduced as compared with the non-GE treated control group (*p* < 0.001). The mice were sacrificed on the 28th day after tumor cell implantation and the tumors were harvested, and the wet weight of the tumor per mouse in each treatment group was recorded. As shown in Figure 3B, the wet weight of the xenograft tumor per mouse was significantly lower in the mice administered GE diet than in the mice fed control diet. This result indicates that dietary GE can inhibit ERα-negative breast cancer *in vivo*.

The second *in vivo* tumor xenograft protocol was designed to evaluate the therapeutic effect of dietary GE and anti-estrogen agent, TAM, on ERα-negative breast cancer based on our previous finding indicating that GE can restore *ERα* reactivation in ERα-negative breast cancer cells. GE diet was given as described previously and TAM was administered two weeks post-injection and maintained release for up to three weeks. As expected, we did not observe any regression in the size of the established tumors after TAM was administered alone due to its poor effect on ERα-negative breast cancer. In the GE-fed mice group, TAM treatment resulted in a significant inhibition of tumor growth rate (*p* <0.001) (Figure 3C). This inhibitory effect on tumor volume began to appear only one week after TAM was administrated and continued until the experiment was terminated. The tumor weight graph in Figure 3D showed the same pattern. To further evaluate the preventive or therapeutic effect of the GE diet alone or combined with TAM treatment on ERα-negative breast xenografts, the inhibition rate on tumor growth (IR) was introduced to compare the efficacy of these treatments. As shown in Table 1, IR in the GE group was significant increased to 50.89% as compared with the non-treatment control (0%) and TAM alone (−1), whereas, most strikingly, IR in the GE plus TAM group was further elevated to 96.6% which meant that most of ERα-negative breast xenografts were inhibited by this novel combination. This result suggests that dietary GE enhances the anti-tumor properties of TAM by re-sensitizing ERα-negative breast cancer to anti-hormone therapy. This finding may provide a new avenue for alternative therapy by combination of dietary GE and anti-hormone therapy for refractory ERα-negative breast cancer.

**Table 1:** Tumor suppression effect of GE and/or TAM on mouse tumor xenografts

**Animal group**	**Diet and treatment**	**BWC**^**a **^**(g, mean ± SD)**	**TV**^**b **^**(mm**^**3 **^**mean ± SD)**	**RTV**^**c **^**(mean)**	**IR**^**d **^**(%)**
Control	Modified AIN-93G diet 7% of corn oil instead of soy oil; no treatment	4.5 ± 1.64	1236.2 ± 195	14.54	-
TAM	Diet is control diet; TAM tablet (25 mg/pellet) was implanted subcutaneously two weeks post-injection	4.9 ± 1.52	1160.5 ± 225.57	14.69	−1
GE	GE diet contains 250 mg genistein/kg of modified AIN-93G diet; no treatment	4.18 ± 1.21	306.9 ± 30.16	7.14	**50.89**
GE + TAM	Diet is GE diet; TAM tablet (25mg/pellet) was implanted subcutaneously two weeks post-injection	4.38 ± 1.46	24.33 ± 4.04	0.45	**96.9**

a. Body weight change (BWC) = (BW on sacrificing day)-(BW of experiment initiation); b. Tumor volume (TV) = (length × width^2^) × 0.532; c. Relative Tumor volume (RTV) = (TV on sacrificing day)/(TV on day 1 of injection); d. Inhibition rate on tumor growth (IR) = {1 - (mean RTV of the treatment group)/(mean RTV of the control group)} · 100.

### Dietary GE increased tumor latency and prevented breast cancer development in spontaneous breast cancer mouse model

To further evaluate the prevention effect of GE treatment as well as its impact on subsequent TAM therapy on ERα-negative breast cancer, we have introduced a spontaneous breast cancer model, C3(1)-SV40 Tag transgenic mouse, in our study. As shown in Figure 3E, GE diet significantly increased mean tumor latency (*p* < 0.001) and reduced 55.56% of breast tumor incidence by 20 wks of age since almost 100% of C3(1)-SV40 Tag mice develop spontaneous breast tumors before 20 wks.

We next sought to study whether mice could respond to TAM treatment to determine the potential interactions between early dietary GE treatment and tumor re-sensitizing to anti-hormone therapy when ERα-negative breast tumor was initiated. We observed tumor growth by measuring tumor volumes in four treatment groups up to 6 weeks when tumor size reached limitation of maximal growth. As shown in Figure 3F, spontaneous tumor growth was only slightly inhibited after TAM treatment, but was significantly reduced by GE treatment. Moreover, GE-fed mice exhibited excellent response to TAM treatment and tumor growth rate was dramatically reduced compared to the other three groups after three-weeks TAM treatment (Figure 3F). These data not only suggest a prevention effect of dietary GE on ERα-negative breast cancer development, but more importantly, long-term consumption of GE-rich food such as soybean products may reinforce efficacy of TAM treatment for ERα-negative breast cancer.

### Dietary GE inhibited tumor cell proliferation and increased ERα expression

**Figure 4:**
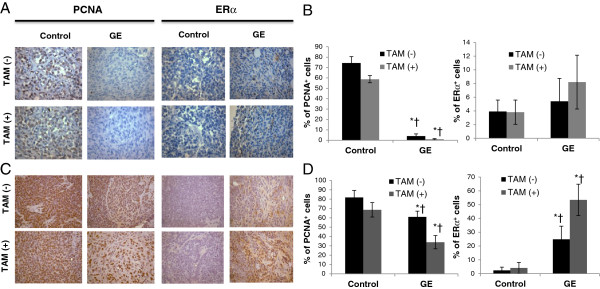
**GE and TAM inhibited the expression of PCNA and increased ERα expression *in vivo*. **Immunohistochemical analysis was performed in tumor samples to detect PCNA-positive cells for proliferation index (left panel) and ERα *in vivo* expression (right panel). **A**) and **B**) PCNA and ERα expression in MDA-MB-231 tumor xenogratfs (*Protocol *1). **C**) and **D**) PCNA and ERα expression in C3(1)-SV40 Tag transgenic mice tumors (*Protocol *2). Immunohistochemical data in terms of percentage of positive cells are presented as mean ± SD from each group. PCNA-positive and ERα-positive cells were counted in 5 different areas of the sections, and data are summarized in terms of percent positive cells from all tumor samples. Representative photograph from one field of each experimental group. Columns, mean; Bars, SD from 5 or 10 mice per group; *, *p *< 0.05 significantly different from control group. †, *P *< 0.05, significantly different from TAM alone group.

Uncontrolled cell proliferation is one of the most important characteristic features of cancer, including breast cancer. We therefore analyzed *in vivo* breast cancer tumors for the potential anti-proliferative property of GE administration. For this purpose, tumor samples were collected and used from the experiment of Figure 3 and subjected to immunohistochemical evaluation. Immunohistochemical detection of PCNA-positive cells in mice xenograft tumors (*Protocol 1*, see Materials and methods) indicated that the percentages of proliferating cells were significantly lower in GE alone and combined with TAM-treated mice tumors than the tumors from the control mice and TAM alone, respectively (Figures 4A and 4B, left panel). Moreover, positive-proliferated cells in the tumor tissue from the combination treatment of GE and TAM were further reduced compared with GE acting alone. In the breast tumors from the mouse prevention model (*Protocol 2*), we found a similar trend as seen in the mouse xenograft tumors (Figures 4C and 4D, left panel) suggesting that GE can prevent breast tumorigenesis via inhibiting tumor cell proliferation and further consolidate anti-tumor effect of TAM treatment. These observations reveal strong preventive and therapeutic efficacy of GE against *in vivo* ERα-negative breast tumor growth and this effect is further enhanced by combination treatment with TAM.

Since the aforementioned studies indicated that GE treatment induced functional *ERα* reactivation *in vitro*, we sought to further investigate whether dietary GE can impact ERα expression that may lead to TAM re-sensitizing to ERα-negative breast cancer *in vivo*. We evaluated ERα expression in mice tumor samples using immunohistochemical analysis. As shown in Figures 4A and 4B, right panel, expression of ERα-positive cells was increased in the xenograft tumor samples from both the GE-fed (5.41%) and GE + TAM-fed groups (8.21%) compared with that of in the control (3.92%) and TAM-fed groups (3.81%), respectively. Furthermore, this effect was more prominent in the mouse prevention model (Figures 4C and 4D, right panel), indicating that long-term consumption of GE diet may lead to a better impact on *ERα* reactivation and TAM treatment enhance this effect. We also found that GE treatment alone can induce a significant increment of *ERα* expression regardless of additional TAM treatment (Figure 4 and Additional file 2C), indicating other potential regulatory mechanisms besides the ER pathway may be involved in GE and TAM-enhanced tumor inhibition on ERα-negative breast cancer.

Taken together, these findings are consistent with our previous studies indicating GE results in increased expression of *ERα* both *in vitro* and *in vivo*, which enhances the efficacy of TAM against ERα-negative breast cancer.

### Expression changes of epigenetic enzymes may affect ERα reactivation in vivo

As we have observed that epigenetic factors may play an important role in regulating GE-induced *ERα* re-expression in ERα-negative breast cells, we next sought to determine whether GE modulated *ERα* expression via epigenetic mechanisms *in vivo*. We therefore chose to evaluate the expression status of DNMT1 and HDAC1 as the most important epigenetic enzymes involving DNA methylation and histone modification accompanied with expression changes of *ERα*. Gene expression status at the protein and mRNA levels in both xenograft and spontaneous breast tumors were detected by western-blot assays and real-time PCR.

**Figure 5:**
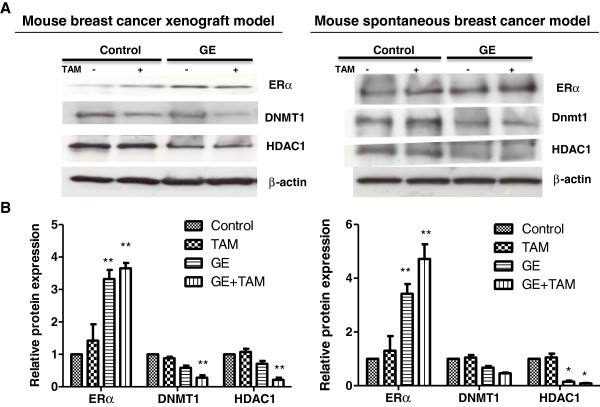
**Protein expression changes of ERα and two epigenetic modulators, HDAC1 and DNMT1 in mice breast tumors. **Left panel, MDA-MB-231 breast tumor xenografts (*Protocol 1*); right panel, C3(1)-SV40 Tag transgenic mouse tumors (*Protocol 2*). **A**) Protein levels of ERα, DNMT1 and HDAC1 in breast tumors using western blot analysis. GE and/or TAM treatments were described in Materials and methods. Representative blots are presented from the independent experiments from all tumors per group with identical results. All the analyses in tumor samples were performed at the termination of the experiment. **B**) Histogram of quantification of the protein levels. Data are in triplicate from three independent experiments and were normalized to actin and calibrated to levels in control samples. Columns, mean; Bars, SD; *, *P *< 0.01, * * *P *< 0.001, significantly different from control.

**Figure 6:**
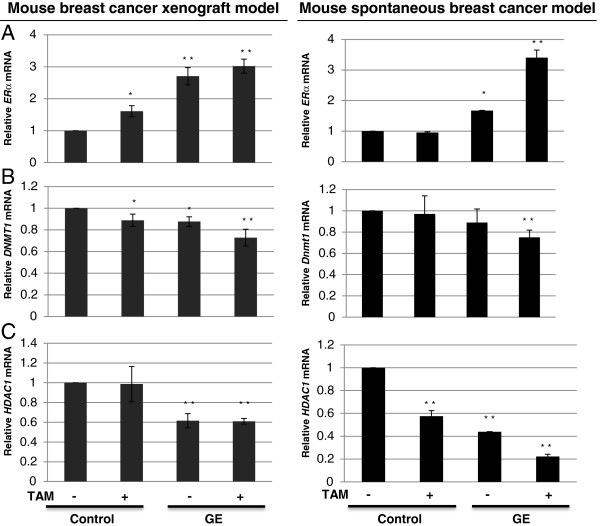
**mRNA expression changes of *ERα*, *HDAC1 *and *DNMT1 *in mice breast tumors. **Left panel, MDA-MB-231 breast tumor xenografts (*Protocol 1*); right panel, C3(1)-SV40 Tag transgenic mouse tumors (*Protocol 2*). **A**) mRNA expression of *ERα*. **B**) mRNA expression of *DNMT1*. **C**) mRNA expression of *HDAC1*. mRNA expression in breast tumors was analyzed by quantitative real-time PCR. GE and/or TAM treatment were described in Materials and methods. All the analyses in tumor samples were performed at the termination of the experiment. Columns, mean; Bars, SD; *, *P *< 0.05, significantly different from control; * * *P*< 0.01, significantly different from control.

As indicated in Figure 5A left panel, first row and Figure 5B left panel, GE treatment alone and combination treatment of GE and TAM induced significant ERα protein re-expression in mice breast xenografts (*p* <0.001). Consistently, *ERα* mRNA level (Figure 6A left panel), was significantly increased in GE-fed alone/combination mice xenografts compared with control group (*p* <0.05), especially in the presence of GE (*p* <0.01). Although the mRNA level of *ERα* treated by TAM alone in mouse xenografts showed significant increased expression in Figure 6A left panel, the protein level did not show similar change as indicated in Figure 4B and Figure 5B left panel. In addition, our *in vitro* result (Additional file 2C) and results in spontaneous mouse models (Figure 4D and Figure 5B right panel) did not show similar effects, which indicates that TAM treatment alone may not be able to induce *ERα* expression and this solo increment of *ERα* may involve certain post-translational regulation depending on different model system or cell types. ERα protein expression was significantly increased in the spontaneous breast tumors with GE treatment alone or combined GE and TAM treatment as compared to the control group (Figure 5A right panel, first row and Figure 5B right panel), which is consistent with its expression at the mRNA level (Figure 6A right panel).

In terms of the expression status of DNMT1 and HDAC1 (Figures 5, 6B and 6C), dietary GE caused a gradual reduction of the expression of these enzymes at the protein and mRNA levels in both tested mouse models, especially when GE and TAM were acting together (*p* <0.01). These results indicate that epigenetic mechanisms may contribute to GE-induced *ERα* re-activation leading to increased sensitivity of TAM therapy toward intractable ERα-negative breast cancer.

### Epigenetic enzymatic activities changes in response to GE and TAM treatment in vivo

**Figure 7:**
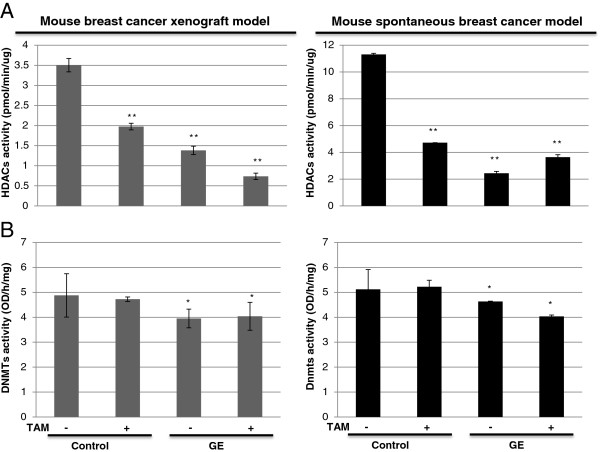
**Epigenetic enzymatic activities in response to dietary GE and/or TAM treatment in mice breast tumors. **Left panel, MDA-MB-231 breast tumor xenografts (*Protocol 1*); right panel, C3(1)-SV40 Tag transgenic mouse tumors (*Protocol 2*). GE and/or TAM treatment were described above. **A**) HDACs enzymatic activity. **B**) DNMTs enzymatic activity. Nuclear proteins from mice breast tumors were extracted as described previously. The HDACs and DNMTs activity assays were performed according to the manufacturer’s protocols. The values of enzymatic activities of HDACs and DNMTs are the means of three independent experiments from all tumors per group. Columns, mean; Bars, SD. *, *P *< 0.05, significantly different from control; * * *P*< 0.01, significantly different from control.

Our observations on expression changes of DNMT1 and HDAC1 indicated that GE alone or combined with TAM treatment led to a significant decrease in expression of these two important epigenetic enzymes (Figures 5, 6B and 6C). We next sought to investigate whether this reduced expression can result in direct enzymatic activities changes *in vivo* that may contribute to epigenetic mechanisms-modulated gene expression alteration such as *ERα* re-activation. We assessed the epigenetic enzymatic activities of HDACs and DNMTs in both xenograft and spontaneous breast tumors. As shown in Figure 7A, both GE and TAM treatment alone and in combination can significantly reduce HDACs activity compared to the control group in the two tested mouse models. In addition, we found that the combination of GE and TAM led to a more prominent reduction than any treatment acting alone in mouse xenografts rather than spontaneous breast tumors, suggesting that GE exposure time could be a key factor influencing TAM-induced epigenetic regulation. However, as to DNMTs activity shown in Figure 7B, only GE treatment caused a slight inhibition suggesting that dietary GE treatment is primarily mediated through histone remodeling rather than DNA methylation, which is consistent with our previous *in vitro* studies. We found that TAM, acting as an anti-hormone drug, may exert its anti-cancer properties by interacting with epigenetic modulators such as DNMTs or HDACs [39]. This may explain our previous results indicating that TAM enhanced GE-induced anti-cancer properties through, at least in part, *ERα* reactivation. TAM may influence epigenetic pathways that facilitate the epigenetic effects of GE leading to *ERα* activation. These results suggest an important synergistic interaction between GE and TAM against ERα-negative breast cancer.

In summary, our results indicate that dietary GE may affect *ERα* expression via modulating epigenetic pathways, especially, histone modification. In addition, dietary GE reinforced TAM-caused anti-cancer effects through increased therapeutic target via up-regulated *ERα* and potential interaction between these two compounds resulting in epigenetic modulations of more relevant genes.

## Discussion

Human breast cancer is phenotypically heterogeneous and the clinical treatment principle of this disease is largely dependent on distinct molecular alterations, for example, the expression status of the nuclear estrogen receptor (ER) [1-3]. ER-positive breast cancers respond to hormonal therapy; however, at least 20% of breast cancer cells that lack of ER expression are more aggressive and have a poor prognosis [3]. Previous work from our laboratory and others has highlighted the restoration of ER signaling through epigenetic pathways for application to a new therapeutic strategy for the ER-negative breast tumors that do not respond to hormone receptor-based treatment such as tamoxifen (TAM) [32].

We started our work on an epigenetic diet, soybean genistein (GE), not only because its proven anti-cancer properties, but also its excellent physiological availability and safety use potentially for clinical transition. It is a therapeutic target worthy of testing GE in those specific classes of breast cancers if *ER* expression is elevated and anti-hormone treatment will be available for the refractory ERα-negative breast cancer. Strikingly, our results showed that GE induced a maximal *ERα* increment at 25 μM in a time-dependent manner (Figure 1A). The concentration of 25 μM GE is equivalent to a maximal daily consumption of soybean product and can also be physiologically attained in blood serum when administrated with a pharmaceutically-available genistein tablet [40], which suggests that this concentration has good bioavailability that could potentially apply for *in vivo* studies. Our further studies revealed a synergistic effect of GE treatment combined with an epigenetic modulator, the HDAC inhibitor TSA, suggesting that this combination may trigger a reciprocal relationship and histone regulations are likely to contribute to favorably stimulate *ERα* expression. Active ERα signaling transports hormone estrogen signal from the outside space of the cell membrane into the nucleus to regulate cellular proliferation and differentiation in normal mammary glands as well as the malignant progression of breast cancer [4,5]. Our further observation of a positive response to hormone signal E_2_ and E_2_ antagonist, TAM, suggests a functional *ERα* re-expression and restoration of *ERα* signal transduction in GE-treated ERα-negative breast cancer cells. These findings should have practical importance since endocrine therapies are usually designed to block ER function, and GE may be applied for sensitization of ERα-negative breast cancer cells to anti-hormone therapy.

The bioactive dietary component, for instance, green tea EGCG {(−)-epigallocatechin-3-gallate}, has been shown to activate *ER* expression via epigenetic control *in vitro*[32,41]. We speculated that GE may impact *ER* gene expression through similar epigenetic regulations as EGCG. Our studies revealed that histone modification may play a more important role in regulating GE-modulated *ERα* restoration rather than DNA methylation. Histone modifications affect the basic structure of the chromatin unit, the nucleosome, and histone acetylation or deacetylation changes are considered to be the most prevalent mechanisms of histone modifications [42]. Histone acetylation results in an open chromatin structure leading to active gene transcription. We found that treatment with GE, especially GE combined with TSA, increased the histone acetylation level in the *ERα* promoter region, which could be considered as an important contributor for *ERα* reactivation. Although we did not find any methylation status changes in the *ERα* promoter region by GE treatment, *ERα* can be regulated by numerous cis-regulatory elements located upstream of the coding sequence of *ERα* and DNA methylation may influence these elements leading to *ERα* expression change. In addition, altered DNMTs enzymatic activities and protein expression *in vitro* and *in vivo* in response to GE treatment indicate that DNA methylation may affect *ERα* expression through DNMT-involved transcription regulation, suggesting DNA methylation may also play a role in GE-induced *ERα* activation.

We further tested this hypothesis by using two different mouse models, the orthotopic and spontaneous breast tumor mouse models, aiming at treatment and preventive effect of dietary GE, respectively. We initiated our *in vivo* studies by applying single GE treatments rather than GE/TSA combination in mice diet due to potential toxicity of TSA in previous clinical studies [43,44]. Our *in vivo* mouse studies supported our *in vitro* results suggesting that dietary GE can not only prevent ERα-negative breast cancer development, but also greatly enhance the anti-cancer capacity of TAM treatment. Although GE treatment alone can cause significant tumor growth retardation which may be due to its proven activities such as anti-oxidation and induction of apoptosis, our observations show more important clinical correlations when a conventional anti-hormone treatment such as TAM is administered with GE. We noticed that short-term dietary GE administration only induced a limited increase of *ERα* expression in mouse xenografts, which may suggest a potential quantity control of *ERα* expression by GE since this slight *ERα* increment may resensitize TAM treatment but avoid uncontrolled cell proliferation caused by *ERα* over-expression [45]. Furthermore, long-term consumption of GE diet resulted in a relatively large elevation of *ERα* expression in spontaneous breast tumors suggesting a protective effect of GE for prevention of ERα-negative breast cancer and a subsequent increment of TAM sensitivity by early reversing *ERα* signaling. Our further observations on selective epigenetic gene expression profiles as well as key epigenetic enzymatic activities in mouse tumors indicate that epigenetic control also plays an important role during this process, which is consistent with our findings in the cellular system. These data provide an important clinical implication for the beneficial effects of dietary soybean products on chemoprevention of refractory hormone-resistant breast cancer and favorable interaction with the treatment benefits of anti-hormone therapeutic agents.

## Conclusions

Collectively, our findings suggest an important role of soybean genistein (GE) on the resensitization to anti-hormone therapy of TAM by inducing functional *ERα* reactivation in ERα-negative breast cancer through, at least in part, epigenetic mechanisms. The concentration of GE we used for *in vitro* and *in vivo* studies is safe and physiologically available, which could be potentially used in future human studies. The involvement of epigenetic control of GE in regulating *ERα* expression is novel and may provide new avenues for potential epigenetic therapy in ERα-negative breast cancer. Moreover, the subsequent function of GE in prevention breast cancer and resensitizing the traditional TAM treatment via *ERα* is very important since it may provide new preventive and therapeutic strategies for ERα-negative breast cancer as well as refractory triple-negative breast cancer (ER, PGR and HER2/neu negative). In conclusion, our findings provide useful observations relevant to clinical prevention and therapeutic application for *de novo* hormone-resistant breast cancer patients. It provides novel preventive and therapeutic approaches targeting *ERα* reactivation through selective consumption of the natural dietary ingredient, GE, combined with anti-hormone therapeutic agents against hormone-resistant breast cancer. Future efforts aimed at human clinical trials are urgently needed to lead the applicability of these novel approaches.

## Abbreviations

GE: Genistein; ER: Estrogen receptor; TSA: Trichostatin A; 5-aza: 5-aza-2’-deoxycytidine; TAM: Tamoxifen; PGR: Progesterone receptor; HDACs: Histone deacetylases; DNMTs: DNA methyltransferases; ChIP: Chromatin immunoprecipitation.

## Competing interests

The authors declare that they have no competing interests.

## Authors’ contributions

Conceived and designed the experiments: YL and TOT; Performed the experiments: YL, SMM, SNP, HC and TMH; Analyzed the data: YL and TOT. Contributed reagents/materials/analysis tools: YL and TOT; Wrote the manuscript: YL; Edited the manuscript: YL, SMM, SNP and TOT. All authors read and approved the final manuscript.

## Supplementary Material

Additional file 1 **GE and TSA synergistically induced *ERα *re-expression in ERα-negative MDA-MB-157 breast cancer cells, but caused no toxicity in normal HMECs cells. A**) Graphic presentation of dose-dependent *ERα *expression by GE treatment. MDA-MB-157 cells were plated in 96-well plates in triplicate and exposed to various concentrations of GE for 3 days. **B**) *ERα *expression changes by the combined treatment of GE with 5-aza (left) and TSA (right). The MDA-MB-157 cells were treated with or without either 25 μM GE or 2 μM 5-aza and 100 ng/ml TSA alone or together for 3 days. Control cells were grown in parallel with the treated cells but received vehicle DMSO. Quantitative real-time PCR was performed to measure relative transcription of *ERα*. Data are in triplicate from three independent experiments and were normalized to *GAPDH *and calibrated to levels in untreated samples. **C**) GE and TSA treatment on normal human breast HMECs cells. HMECs cells were treated with 25 μM GE and 100 ng/ml TSA alone or together for 3 days as described above. Cellular viability was measured by MTT assay. Data are in triplicate from three independent experiments and were normalized to levels in control samples. Columns, mean; Bars, SD; *, *P *< 0.05, * * *P*< 0.001, significantly different from control; £, *P *< 0.05, significantly different from GE; †, *P *< 0.05, significantly different from 5-aza or TSA. Click here for file

Additional file 2 **Reactivated *ERα *potentiates the anti-cancer efficacy of GE and TAM. A**) *ERα* presence affects GE and/or TAM-induced cellular growth inhibition. MDA-MB-231 cells were transfected with ERα RNAi for two days and then plated in 96-well plates in triplicate and exposed to various concentrations of GE and/or TAM for another 3 days. Cellular viability was measured by MTT assay. **B**) *ERα *expression verification after *ERα *silencing treatment. MDA-MB-231 cells were treated as described above and parallel mRNAs were collected for *ERα *expression. **C**) *ERα* expression changes in response to GE and TAM treatment. MDA-MB-231 cells were treated with 25 μM GE and/or 1 μM TAM as described in Materials and methods. Quantitative real-time PCR was performed to measure relative transcription of *ERα*. Data are in triplicate from three independent experiments and were normalized to *GAPDH *and calibrated to levels in untreated samples. Columns, mean; Bars, SD; *, *P *< 0.05, * * *P*< 0.01, significantly different from control. Click here for file
