# Supplementary material for: Epigenetic reactivation of estrogen receptor-α (ERα) by genistein enhances hormonal therapy sensitivity in ERα-negative breast cancer
Source: Mol Cancer. 2013 Feb 4;12:9. doi: 10.1186/1476-4598-12-9 (PMC3577460; doi:10.1186/1476-4598-12-9)
Supplement: Additional file 1 — GE and TSA synergistically induced ERα re-expression in ERα-negative MDA-MB-157 breast cancer cells, but caused no toxicity in normal HMECs cells. A) Graphic presentation of dose-dependent ERα expression by GE treatment. MDA-MB-157 cells were plated in 96-well plates in triplicate and exposed to various concentrations of GE for 3 days. B) ERα expression changes by the combined treatment of GE with 5-aza (left) and TSA (right). The MDA-MB-157 cells were treated with or without either 25 μM GE or 2 μM 5-aza and 100 ng/ml TSA alone or together for 3 days. Control cells were grown in parallel with the treated cells but received vehicle DMSO. Quantitative real-time PCR was performed to measure relative transcription of ERα. Data are in triplicate from three independent experiments and were normalized to GAPDH and calibrated to levels in untreated samples. C) GE and TSA treatment on normal human breast HMECs cells. HMECs cells were treated with 25 μM GE and 100 ng/ml TSA alone or together for 3 days as described above. Cellular viability was measured by MTT assay. Data are in triplicate from three independent experiments and were normalized to levels in control samples. Columns, mean; Bars, SD; *, P < 0.05, * * P< 0.001, significantly different from control; £, P < 0.05, significantly different from GE; †, P < 0.05, significantly different from 5-aza or TSA. [file 1476-4598-12-9-S1.pptx]

## Slide 1
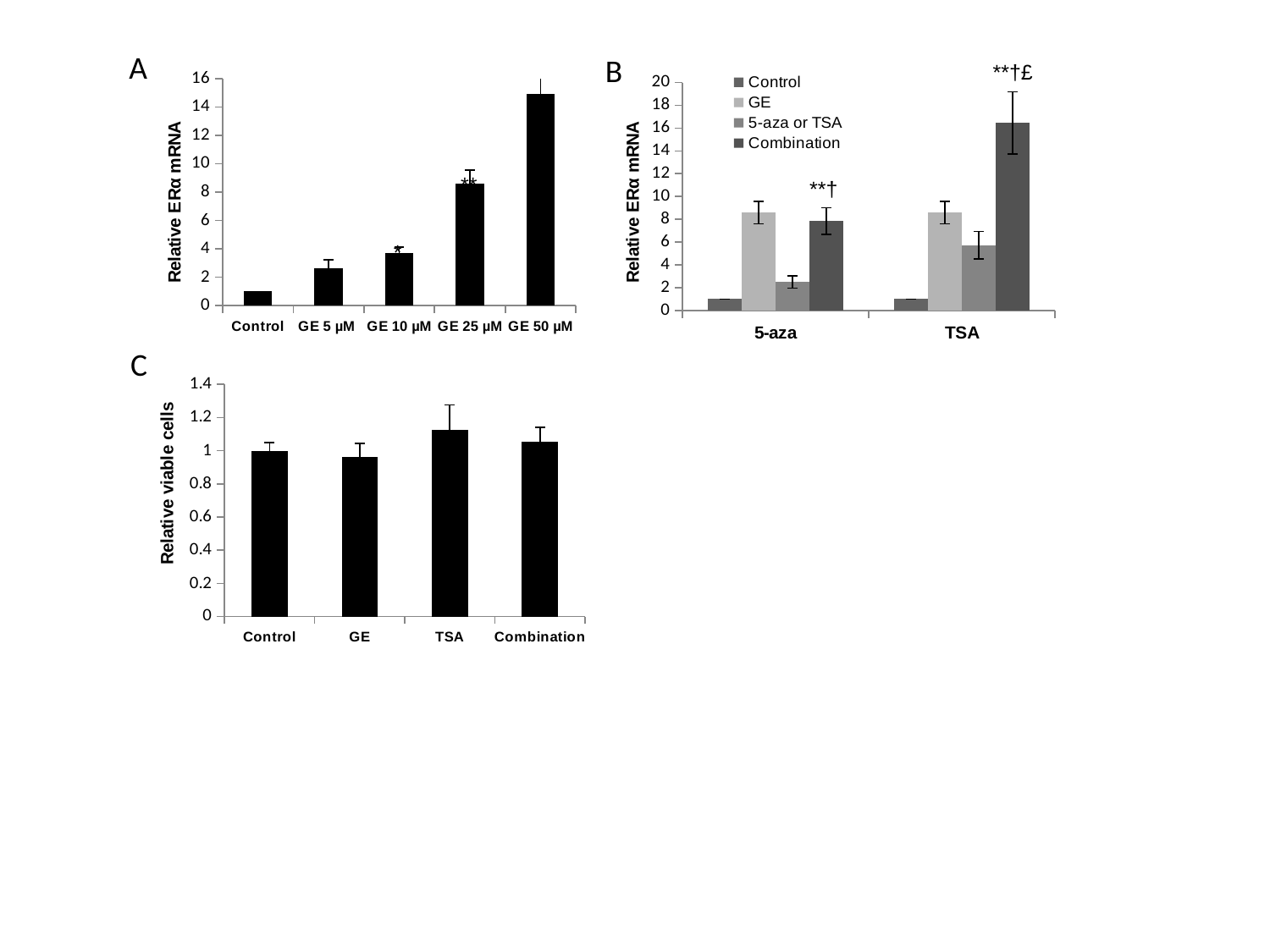

A
B
**†£
### Chart
| Category | Control | GE | 5-aza or TSA | Combination |
|---|---|---|---|---|
| 5-aza | 1.0 | 8.574187700290352 | 2.496661097803224 | 7.835362380695408 |
| TSA | 1.0 | 8.574187700290352 | 5.735820992063322 | 16.449821226497093 |**†
### Chart
| Category | |
|---|---|
| Control | 1.0 |
| GE 5 µM | 2.6026837108838587 |
| GE 10 µM | 3.7063522475614756 |
| GE 25 µM | 8.574187700290352 |
| GE 50 µM | 14.928527864588904 |**
**
*
C
### Chart
| Category | |
|---|---|
| Control | 1.0 |
| GE | 0.963 |
| TSA | 1.126 |
| Combination | 1.054 |
