# Supplementary material for: Epigenetic reactivation of estrogen receptor-α (ERα) by genistein enhances hormonal therapy sensitivity in ERα-negative breast cancer
Source: Mol Cancer. 2013 Feb 4;12:9. doi: 10.1186/1476-4598-12-9 (PMC3577460; doi:10.1186/1476-4598-12-9)
Supplement: Additional file 2 — Reactivated ERα potentiates the anti-cancer efficacy of GE and TAM. A) ERα presence affects GE and/or TAM-induced cellular growth inhibition. MDA-MB-231 cells were transfected with ERα RNAi for two days and then plated in 96-well plates in triplicate and exposed to various concentrations of GE and/or TAM for another 3 days. Cellular viability was measured by MTT assay. B) ERα expression verification after ERα silencing treatment. MDA-MB-231 cells were treated as described above and parallel mRNAs were collected for ERα expression. C) ERα expression changes in response to GE and TAM treatment. MDA-MB-231 cells were treated with 25 μM GE and/or 1 μM TAM as described in Materials and methods. Quantitative real-time PCR was performed to measure relative transcription of ERα. Data are in triplicate from three independent experiments and were normalized to GAPDH and calibrated to levels in untreated samples. Columns, mean; Bars, SD; *, P < 0.05, * * P< 0.01, significantly different from control. [file 1476-4598-12-9-S2.pptx]

## Slide 1
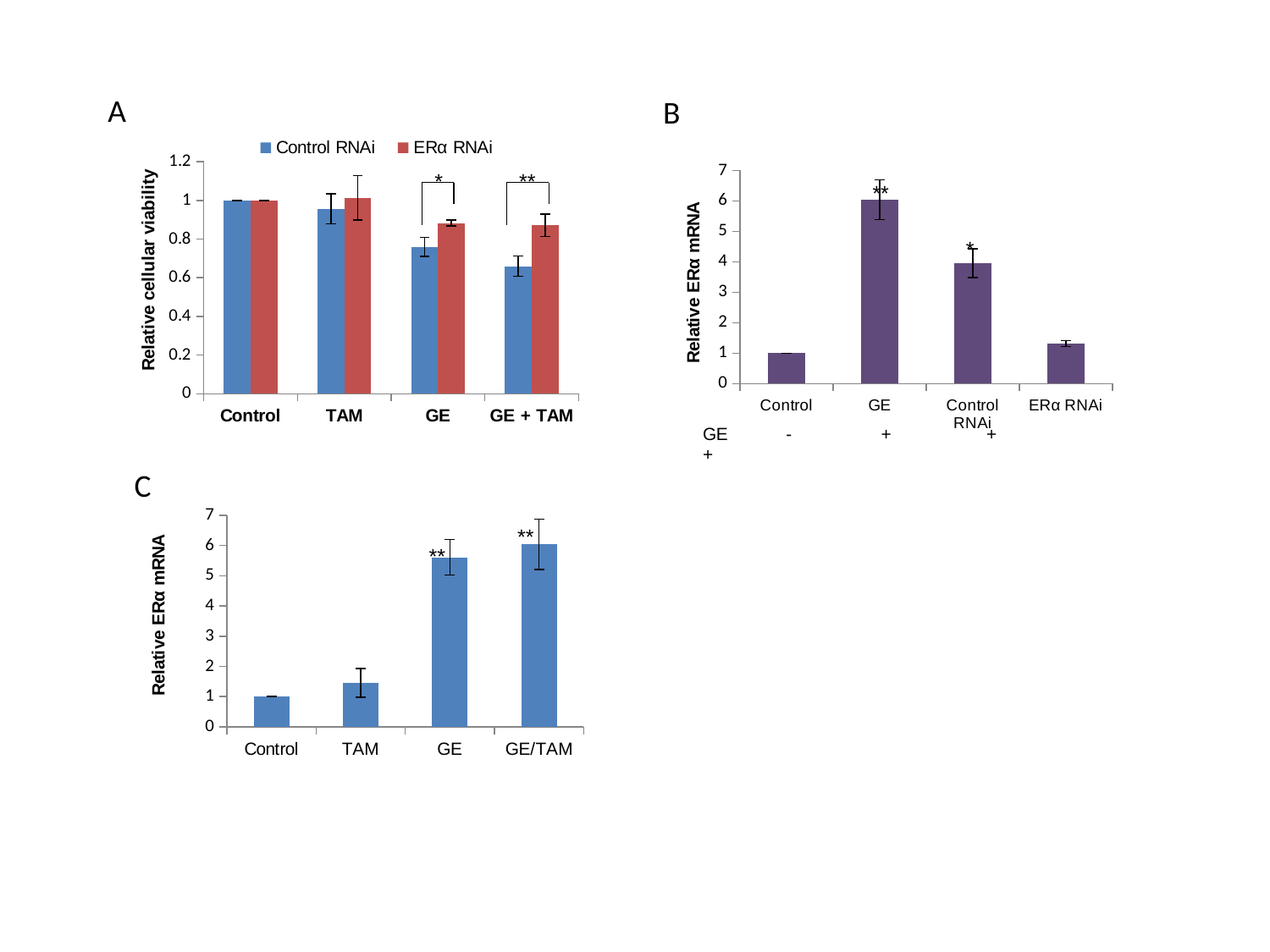

A
B
### Chart
| Category | Control RNAi | ERα RNAi |
|---|---|---|
| Control | 0.9999999999999999 | 0.9999999999999999 |
| TAM | 0.9570753478603307 | 1.0139521640091116 |
| GE | 0.760173273825151 | 0.8832574031890661 |
| GE + TAM | 0.66 | 0.8721526195899771 |*
**
### Chart
| Category | |
|---|---|
| Control | 1.0 |
| GE | 6.04 |
| Control RNAi | 3.96 |
| ERα RNAi | 1.31 |**
*
C
### Chart
| Category | |
|---|---|
| Control | 1.0 |
| TAM | 1.454 |
| GE | 5.61 |
| GE/TAM | 6.04 |**
**
